# Acceptability of Serious Games in Pediatric Asthma Education and Self-management: Pilot Study

**DOI:** 10.2196/33389

**Published:** 2022-04-07

**Authors:** Nicole Silva-Lavigne, Alena Valderrama, Sandra Pelaez, Myriam Bransi, Fabio Balli, Yannick Gervais, Thomas Gaudy, Sze Man Tse

**Affiliations:** 1 Faculty of Medicine University of Montreal Montreal, QC Canada; 2 Health Promotion Center Sainte-Justine University Hospital Center Montreal, QC Canada; 3 Breathing Games Association Geneva Switzerland; 4 Research Centre Sainte-Justine University Hospital Center Montreal, QC Canada; 5 School of Kinesiology and Physical Activity Sciences Faculty of Medicine University of Montreal Montreal, QC Canada; 6 Faculty of Medicine Laval University Quebec, QC Canada; 7 Department of Pediatrics Centre mère-enfant Soleil of Quebec City University Hospital Center Quebec, QC Canada; 8 Concordia University Montreal, QC Canada; 9 Division of Respiratory Medicine Department of Pediatrics Sainte-Justine University Hospital Center Montreal, QC Canada

**Keywords:** asthma, pediatrics, video games, eHealth, self-management

## Abstract

**Background:**

Asthma is the most common chronic pediatric disease. Despite existing tools to manage asthma, 40%-55% of children with asthma experience uncontrolled asthma. Serious games (SGs) represent a novel approach in promoting asthma education and self-management for children.

**Objective:**

In this qualitative pilot study with an embedded quantitative design, we aim to use focus groups and questionnaires to describe the perceived role of SGs in different aspects of asthma self-management by children and their parents. These aspects include asthma perception and knowledge, the impact of asthma and barriers to asthma self-management, and the support system for asthma self-management.

**Methods:**

A total of 5 children with asthma and their parents were invited to participate in an organized gaming session. Children and their parents completed a pregaming questionnaire on their medical history and asthma knowledge. Then, they were invited to test 4 original SG prototypes, after which the children answered a postgaming questionnaire on their asthma knowledge and perception of the SGs. Children and their parents subsequently participated in parallel focus groups, which were video-recorded or audio-recorded, transcribed verbatim, and analyzed by reaching consensus among members of the research team.

**Results:**

The mean age of the children was 10.3 (SD 1.5) years, with 20% (1/5) of the children being male. Qualitative data from the transcripts were coded into three separate domains: asthma self-management perception and knowledge, impact of asthma and barriers to asthma self-management, and support system for asthma self-management. We specifically explored the perceived roles of SGs within each domain. A key takeaway message was identified for each of these three domains: heterogeneity of asthma knowledge and the ability of SGs to encourage knowledge transfer through games, consequences and limitations of asthma and the ability of SGs to allow for identification and management of real-life situations through games, and insufficient support system and the ability of SGs to encourage playing with others for support and shared knowledge.

**Conclusions:**

Our pilot study explored the role of SGs in the self-management of asthma, as perceived by children and their parents. Our findings support the acceptability of SGs in asthma education and self-management in pediatrics and the necessity for future development in this field.

## Introduction

### Background

Asthma, characterized by persistent inflammation of the airways and limited airflow, is the most common chronic pediatric disease [1]. Worldwide, studies consistently report that 40%-55% of children with asthma have uncontrolled asthma in the outpatient setting, defined as frequent respiratory symptoms and exacerbations necessitating the use of health care resources [2-5]. Poor asthma control can compromise long-term lung function and increase the risk of exacerbations, which can negatively impact a child’s school attendance and participation in activities [1,6-8] and is a major source of stress for families [1,9]. Despite existing tools for asthma education such as targeted one-on-one asthma education and accessible web-based or paper-based asthma information, inefficient knowledge transfer and poor adherence to prescribed therapy remain major contributors to poor asthma control [1,6,10,11]. For example, studies have shown that 50% of adults and children who are prescribed daily asthma medications do not take them as recommended [1,11], and that poor inhaler technique is common, with 70%-80% of patients using their inhalers incorrectly [1]. A study focused on patients’ perspective of taking long-term asthma controller medication reported various barriers to adherence, including doubts about asthma severity, fears of addiction, and limited knowledge [12]. Thus, novel tools for asthma education are needed to ensure adequate asthma control.

Recent and ongoing technological advances have allowed the field of eHealth to prosper and evolve greatly. eHealth is the cost-effective use of technologies in various fields of health, namely, health education [13]. The Chronic Care Model (CCM), a validated framework for the management of patients with chronic illnesses, has recently been updated to create the eHealth Enhanced CCM (eCCM), highlighting the potential benefits that eHealth could have for such individuals [14]. This model presents various components that impact the management of chronic illnesses and eHealth applications, which can contribute to improved outcomes [14]. More precisely, it has been suggested that eHealth tools such as telehealth and mobile health apps could be useful in self-management support, resulting in more engaged and empowered patients [14]. Moreover, the eCCM includes eHealth education as a component of chronic care management, stressing the importance of health literacy and eHealth training [14]. The eCCM equally highlights the importance of having a patient who is informed and activated and a practice team that is prepared and proactive, with productive interactions between these 2 actors also contributing to improved outcomes [14].

### Previous Work

Although the eCCM focuses primarily on implementing eHealth tools, such as the use of internet for health information, mobile health, telehealth, electronic health records, and personal health records or patient portals, it does not specifically address the role of serious games (SGs) in health or in chronic illness management [14]. SGs are games that impart real-world skills, knowledge, or attitudes to the user through play [15]. They represent a cost-effective, accessible, unique, and dynamic approach within eHealth that can positively impact self-management support and eHealth education, which are 2 key components of the eCCM, through behavioral change by including simulation and management activities [14-17]. A systematic review of SGs in asthma education revealed that 90% of the children enjoyed them, and that most studies resulted in an improvement in the child’s knowledge about asthma [15]. However, the vast majority of the games failed to demonstrate significant changes in behavior and clinical outcomes, possibly because they were directed uniquely at the children and not their parents, despite parents playing a major role in the treatment of their children [15]. Furthermore, previous SGs mainly focused on information delivery rather than simulations of real-life scenarios, which are more likely to promote behavior change. Finally, existing SGs are web-based or desktop computer–based, and their effectiveness may be enhanced if they are designed as accessible mobile apps [15].

### Goal of This Study

To evaluate how SGs can be integrated into the asthma management framework, we build and, in a pilot study, evaluate 4 open-source SG prototypes focused on recognizing asthma triggers and symptoms and taking appropriate actions during an asthma exacerbation or as part of control therapy. These games are developed to provide a novel technique to promote self-management of asthma in children, which is a key aspect of asthma action plans [2,18]. They represent different game genres, including action, role-playing, and simulation. This pilot study aims to evaluate the acceptability of these original bilingual (French and English) SGs and gather feedback to guide their further development by answering the following research question: what are the children’s and their parents’ perceptions of the role of SGs in the self-management of asthma?

## Methods

### Study Design

A qualitative study with a consensual qualitative design and an embedded quantitative component [19,20] was used to explore children’s and their parents’ perceptions of the role of SGs in different aspects of asthma self-management. We chose a consensual qualitative design because we emphasized the agreement between members during focus groups and within the research team.

### Ethics Approval

The study obtained approval from the research ethics boards of both the Sainte-Justine University Health Center (ID 2019-2075) and Concordia University (ID 30010592). Written informed consent and written or oral assent for the study and video-recording or audio-recording were obtained from the parents and from age-appropriate children, respectively.

### Participants and Procedures

We identified children meeting the eligibility criteria through the appointment list and a chart review, and the families were consecutively approached by the research team for participation at the respiratory medicine or asthma clinic of the Sainte-Justine University Health Center, a pediatric tertiary care center. Participants were children (1) aged 8-12 years inclusively, (2) with physician-diagnosed asthma, (3) who were on a daily controller medication, and (4) who understood and spoke French or English. We excluded children with chronic conditions other than asthma, such as cardiovascular diseases, neuromuscular disorders, or developmental delay. The targeted sample size of this study was 14 children and one of each of their parents. This sample size was based on previous literature using focus groups in pediatric health care research [21-23], the expertise of a qualitative researcher on our team, and the feasibility for a pilot study.

### Data Collection

#### Pregame and Postgame Questionnaires

Pregaming questionnaires were administered separately to both parents and children upon their arrival. The pregaming questionnaires included questions pertaining to previous gaming experience (children), asthma perception (children and parents), asthma knowledge (children and parents), and medical history of the child (parents). Asthma perception was assessed through several statements about general health and asthma using a Likert scale. The asthma knowledge questionnaire included true or false questions pertaining to their knowledge of asthma. In the absence of a well-validated asthma knowledge questionnaire, we created this questionnaire based on previous studies [24-26]. The final questions were reviewed by the research team, which includes a pulmonologist, a pediatrician, and a public health specialist, for their relevance to childhood asthma. The asthma knowledge questions were identical for both the pregaming and postgaming questionnaires to evaluate the knowledge transfer achieved through the SGs. We asked the children to play the 4 SGs on a provided laptop for a total duration of approximately 60 minutes. In addition, we encouraged the parents to explore the games themselves, either on their own or by playing with their children. A description of the games is provided below (Table 1). After each game, we asked the children to answer a web-based survey based on a Likert scale, about their general opinions of the game. After the gaming experience, the children completed a postgaming questionnaire, which included questions on the strengths and weaknesses of the games and the asthma knowledge questionnaire.

**Table 1:** Description of the 4 games used in this study.

Name of the game	Game description	Educational objectives	Time allotted to play (minutes)
Asthmonautes	A game in which the child navigates through different scenarios and interacts with 9 characters to learn about asthma symptoms and management [27]	Understand asthma symptoms and management	30
Lung Launcher	A game in which the character encounters different asthma triggers (customizable to the child) and the child must find the correct preventive method to address each trigger [28]	Identify asthma triggers and learn how to address them	4
Asthma Heroes	A game in which the player interacts with several characters to learn about their symptoms, treatment, and context and collects objects to help them manage their asthma [29]	Understand asthma symptoms and management	30
Bloïd	A game in which the player uses a breath-actuated sensor as an input device to guide a spacecraft and destroy meteorites in its path [30]	Be aware of own breathing	4

#### Focus Groups and Interviews

We conducted semistructured focus groups with children and parents separately. Individual interviews were conducted on one of the study days, as there were only 2 children and 2 parents present. Of note, the interviewers were not involved in the clinical care of the participants and were unfamiliar to them. The topic guides used to collect data considered the inclusion of probing questions that were used accordingly to obtain more detailed responses. The topic guide for the children’s focus group included themes such as asthma in general, gaming experience, practical implications the games could have on their health including benefits and risks, and the potential of playing SGs at home. The topic guide for the parents’ focus group revolved around asthma management and challenges, available resources to help overcome their challenges, and the acceptability of SGs in health. The interview and focus group times ranged from 16-35 minutes. We aimed to achieve consensus among the participants during the focus groups.

### Data Analysis

We used the predetermined questionnaires for the quantitative part of the study and predetermined discussion topics to evaluate the topics of interest qualitatively. Then, the recordings were transcribed verbatim. For confidentiality purposes, all personal information was removed from the transcripts and participants were allotted a study identification number.

We described the participants’ characteristics and information pertaining to their asthma. The analysis of the qualitative data was guided by the eCCM [14], which highlights important aspects of self-management and eHealth education in chronic illnesses. During the analyses, the members of the research team reached consensus to ensure validity and coherence of the results [19]. Specifically, the analytical process was divided into three major steps, including segmenting data from the transcripts into domains, abstracting data within the domains into core ideas, and performing a cross-analysis to develop themes across participants, which were agreed upon by the research team [20]. We used the software MAXQDA (version 12; VERBI Software) to support data analysis.

To ensure the validity of our study, we implemented various verification techniques throughout the analytical process [31]. By using multiple researchers for data analysis and different data collection methods (eg, questionnaires and focus groups), we were able to achieve analyst triangulation and methods triangulation, respectively, thereby ensuring a consensus among team members and maximizing the credibility and confirmability of our findings. In addition, peer debriefing was used to further establish credibility. Confirmability was further achieved by maintaining an audit trail and ensuring reflexivity by reporting biases, using multiple researchers, and using interviewers who are not involved in the care of the participants. Finally, we described the themes that emerged in this study by using quotes from the participants and their parents as evidence. As the individual interviews and group discussions were conducted in French, the quotes were translated to their English equivalents.

## Results

### Study Participants

The targeted sample size of this pilot study was 14 children and one of each of their parents. We approached 37 potential participants and 14 (38%) families agreed to participate. Reasons for declining participation included lack of time, living far from the hospital, and scheduling conflicts. On the day of the focus groups, owing to unpredictable cancellations (extreme weather and scheduling conflicts), the final sample size was 36% (5/14) of the children and 6 parents (2 parents were present for one of the children). We invited participants to attend one of the 2 half-day sessions organized at the Sainte-Justine University Health Center.

Baseline characteristics of the participants are presented in Table 2. The mean age of the participants was 10.3 (SD 1.5) years, and only 20% (1/5) of the participants were male. None of the participants had any asthma-related hospitalizations or emergency department visits during the 12 months before the study. The average time spent on playing video games each day was 30-60 minutes for 40% (2/5) of the participants. Mobile phones and different consoles were found to be the most used devices for gaming, with 60% (3/5) of the participants using each device.

**Table 2:** Baseline characteristics of the children with asthma included in this analysis (N=5).

Characteristics		Values
Age (years), mean (SD)		10.3 (1.5)
Sex (male), n (%)		1 (20)
Parental report of age of asthma diagnosis (years), mean (SD)		1.9 (1)
Child report of age of first asthma exacerbation (years; for nonmissing data; n=3), mean (SD)		2.2 (0.8)
Asthma-related hospitalization during the past 12 months, median (IQR)		0 (0)
**Time spent on playing video games, n (%)**		
	Never	1 (20)
	0-30 minutes	1 (20)
	30-60 minutes	2 (40)
	60-90 minutes	0 (0)
	90-120 minutes	0 (0)
	>2 hours per day	1 (20)
**Device used to play games, n (%)**		
	Mobile phone	3 (60)
	Tablet	2 (40)
	Computer	2 (40)
	Console	3 (60)

### Analysis of Qualitative Data

**Figure 1:**
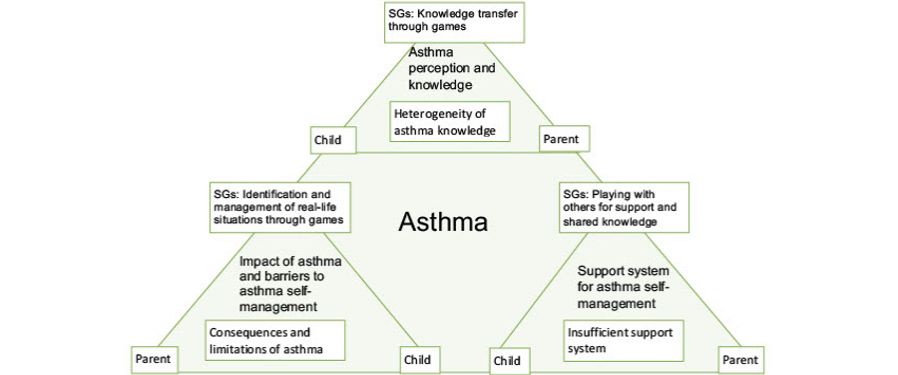
Visual representation of the main results obtained in this study. Our results demonstrated a triangular relationship between a child with asthma, their parent, and the potential role of serious games (SGs) on the 3 domains of asthma management evaluated in this study. The major overarching issues identified during discussions with parents and children, respective to each domain, are also illustrated.

The analysis of the transcripts from the individual interviews and group discussions occurred in 3 major steps and was guided by the following research question: what are the children’s and parents’ perceptions of SGs in the self-management of asthma? The transcripts were analyzed by a primary research team composed of 4 coders (NS, AV, FB, and SMT). Transcripts were first coded into 3 major domains, or topic areas, by one of the coders (NS) and then, verified by the remaining coders on the research team (AV, FB, and SMT). The domains reflected various components of the perceived reality of asthma by children and parents. They included (1) asthma self-management perception and knowledge, (2) impact of asthma and barriers to asthma self-management, and (3) support system for asthma self-management. The data in each domain were abstracted independently into core ideas by 4 coders (NS, AV, FB, and SMT), focusing on the role of SGs within each domain, as perceived by children and parents. Then, the 4 coders worked together to perform a cross-analysis of the core ideas within the domains to generate common themes on the roles of SGs. The team discussed until consensus was achieved. Analysis was guided by the eCCM. After analysis, a visual model was created, illustrating the key interactions between asthma and SGs, as perceived by children and parents (Figure 1). This model complements the original eCCM model by integrating key concepts pertaining to pediatric care and the role of SGs.

### Themes Identified From Interviews and Discussions

#### Overview

Table 3 shows the themes identified from the interviews and discussions.

**Table 3:** Study domains and corresponding themes identified from interviews and discussions with children and parents.

Domain	Themes	
	Children	Parents
1–The role of SGs^a^ in asthma self-management perception and knowledge	Knowledge transfer achieved through the games	Potential of learning about asthma through games and knowledge transfer Gain of knowledge depends on experience and onset of asthma
2–The role of SGs in addressing the impact of asthma and barriers to asthma self-management	Knowledge on self-management through games	Knowledge on self-management through games Utility of games at asthma diagnosis Games as a possible tool to evaluate ongoing asthma control
3–The role of SGs in the support system for asthma self-management	Asthma awareness through SGs Openness to further discuss asthma with parents through games Interest and utility of playing the games with peers	Importance of parent and child playing together Interest and utility of playing the games with peers

^a^SG: serious game.

#### Domain 1: The Role of SGs in Asthma Self-management Perception and Knowledge

This domain included different themes pertaining to the role of SGs in children’s and parents’ asthma self-management perceptions and knowledge. On the basis of the pregaming questionnaires on asthma perception, we already noted some inconsistencies in the children’s perceptions of their ability to manage their asthma. Only 40% (2/5) of the children agreed that they were able to recognize an asthma crisis, whereas 60% (3/5) of the children agreed that they were able to control their asthma adequately, and 80% (4/5) of the children agreed or strongly agreed that their asthma crises could be prevented. Parents were more confident about asthma management, with 80% (4/5) of parents agreeing that they knew how to manage their child’s asthma crises and 80% (4/5) of the parents agreeing or strongly agreeing that they knew when to give each inhaler to their child.

Regarding asthma self-management knowledge, some parents expressed having a divergent perception of asthma severity as compared with that of medical professionals. This is illustrated by the following quote from a parent when questioned whether they were interested in participating in the game with their child:

No, because for us, the only thing that gets our attention is when he isn’t breathing well or when he has allergies. Otherwise, everything else for us is normal. Maybe we lack sensitivity.

parent 03

In addition, parents described the contrasting perceptions of asthma management between parents and their children with asthma, exemplified by the following quote:

Sometimes it’s like she thinks that she takes too many [medications] and she tries to say ‘Oh, I’m going to tough it out, I am still able to breath well’ before taking her pumps. And I tell her, ‘Don’t do that, take it [your pumps] right away’. And she says ‘No, no. I can handle it myself.

parent 08

We also explored data on children’s and parents’ general asthma knowledge in this domain using quantitative data from the asthma knowledge questionnaire. Results from the pregaming questionnaires suggested that participants had a fair amount of asthma knowledge before participating in the study. However, some of the questions in the pregaming questionnaires were unanswered by the participants (Table 4), whereas none of the questions were left unanswered in the postgaming questionnaire. During the discussions with the children, it was also evident that there was heterogeneity in their knowledge about asthma. Some children demonstrated adequate knowledge about asthma, whereas other children expressed a lack of knowledge. Other children lacked knowledge about specific components of their illness, such as asthma management or asthma resources.

On the basis of current scientific knowledge of asthma, several false beliefs were identified among the parents, including that inhalers could lead to dependence or addiction (1/5, 20% agreed), it is not good for children to use the inhaler for very long (3/5, 60% strongly agreed or agreed), children should use asthma medications only when they have symptoms (1/5, 20% strongly agreed; 1/5, 20% neither agreed nor disagreed), inhalers should be used directly in the mouth (2/5, 40% agreed), and controller medications can be used intermittently (3/5, 60% strongly agreed or agreed). In contrast, parents understood other concepts well, with 80% (4/5) of them disagreeing that children with asthma should not exercise or participate in physical education class and that mild asthma attacks can be managed outside of emergency departments. Through the discussions with the parents, we also identified varying degrees of asthma knowledge present among them. Specifically, some parents acknowledged their reluctance to administer prescribed medications unless their child was acutely ill, as illustrated in the following quote:

As long as I can survive without medication, I don’t take any. But, when I see him [the child] suffering sometimes when he has his crises and everything, I am obliged. We even neglected the pump because I am anti-Ventolin because it’s not good for his health in the long term.

parent 03

In contrast, several parents viewed their children’s asthma specialists as important resources in the management of asthma.

Subsequently, we explored how children and their parents perceive SGs in asthma self-management and knowledge and extracted key themes from these data. After analyzing the discussions with the children, we concluded that they were able to identify the game objective correctly and that *knowledge transfer was achieved through the games*, a recurrent theme in the asthma self-management perception and knowledge domain. They were able to create links between the game and real life and understood the steps in medication use and trigger recognition. This knowledge transfer was equally objectified by the results of the postgaming questionnaires (Table 4). In general, the results reflect that after playing the different SGs, the proportion of correct answers increased, notably regarding trigger recognition, medication use, and identification of when the controller or crisis medication must be used.

**Table 4:** Child knowledge of asthma before and after playing the serious games based on true or false questions (N=5).

Child knowledge of asthma–questions	Before game			After game		Discrepancy
	Correct answers, n (%)	Nonmissing data for pregaming questionnaire, n (%)	Correct answers, n (%)		Discrepancies between questionnaires, n (%)	
Lots of children have asthma	5 (100)	5 (100)	5 (100)		0 (0)	
People with asthma can drink milk and eat yogurt	5 (100)	5 (100)	5 (100)		0 (0)	
Having the flu can cause an asthma attack	2 (50)^a^	4 (80)	4 (80)		2 (40)	
Smoking is OK for people with asthma	5 (100)	5 (100)	5 (100)		0 (0)	
People with asthma become hooked on their asthma drugs (cannot get off them)	0 (0)	3 (60)	1 (20)		1 (20)	
If you have asthma now, you will have asthma forever	4 (80)	5 (100)	5 (100)		1 (20)	
An asthma attack is caused by redness and swelling in the airways of the lungs	3 (75)^a^	4 (80)	4 (80)		1 (20)	
Most children with asthma are smaller than other children	4 (100)^a^	4 (80)	5 (100)		1 (20)	
Asthma can be spread from person to person	5 (100)	5 (100)	5 (100)		0 (0)	
Medicines that keep asthma from happening should be taken every day	5 (100)	5 (100)	5 (100)		0 (0)	
The blue puffer (inhaler) should be used when a person has an asthma attack	4 (80)	5 (100)	5 (100)		1 (20)	
Asthma happens more at night	3 (60)	5 (100)	3 (60)		0 (0)	
An asthma attack can happen suddenly without warning	5 (100)	5 (100)	5 (100)		0 (0)	
When asthma is OK, all medicines can be stopped	4 (80)	5 (100)	4 (80)		0 (0)	
With the right treatment, a child with asthma can live a normal life	4 (100)^a^	4 (80)	5 (100)		1 (20)	
Children with asthma can play sports	4 (80)	5 (100)	4 (80)		0 (0)	
The orange inhaler controls asthma	4 (80)	5 (100)	5 (100)		1 (20)	
The blue inhaler helps with an asthma crisis	5 (100)	5 (100)	5 (100)		0 (0)	
The blue inhaler opens the airways in the lungs	3 (60)	5 (100)	5 (100)		2 (40)	
The orange inhaler prevents asthma crises	4 (100)^a^	4 (80)	5 (100)		1 (20)	
The blue inhaler helps the breathing during an asthma crisis	5 (100)	5 (100)	5 (100)		0 (0)	

^a^Sample size, n=4, owing to missing data.

The role of SGs on asthma perception and asthma knowledge as perceived by the parents was identified using the asthma perception questionnaire before playing the SGs and through the discussions with the parents. The questionnaire revealed that 40% (2/5) of parents agreed that health-related video games can help their child understand or manage their asthma, whereas the remaining parents neither agreed nor disagreed. Moreover, 100% (5/5) of the parents strongly agreed or agreed that they would accept that their child plays health-related video games at home. The themes extracted from the discussions with the parents illustrated similar results. Similar to children, the theme *potential of learning about asthma through games and knowledge transfer* was identified by the parents. This theme is illustrated by the following quote from a parent, concerning how one of the SGs could be designed to show the level of respiratory distress:

With the game, I think that she [their child] would realize ‘Oh no, I think that maybe I should take it [medication] as soon as possible’ rather than waiting.

parent 08

In addition, parents expressed that the *gain of knowledge depends on experience and onset of asthma*, with some parents indicating that the incentive to play may be lost after knowledge is gained from the games.

#### Domain 2: The Role of SGs in Addressing the Impact of Asthma and Barriers to Asthma Self-management

This domain included different themes pertaining to the roles of SGs in addressing the impact of asthma and barriers to asthma self-management, as expressed by children and parents. Regarding the impact of asthma, the daily burden of asthma was a recurrent topic expressed by children. This is illustrated by the following quotes:

For me it’s also sports. I tire out before the others.

child 08

We are in the middle of a game and I have to stop. I have to stop to take my pumps. Sometimes I’m scared that I’m a nuisance to my team.

child 09

Although the children recognized the importance of medications in controlling their asthma, they also identified poor adherence as a barrier to asthma self-management.

The impact of asthma and barriers to asthma self-management were also explored with the parents. Parents also referred to the daily burden of asthma on their child and family, as illustrated by the following quote:

She spends every night out of breath. She is always gasping for air. She is always congested. There is no specific season that she is suffering, but it’s almost every day. We almost always go to see the doctor. We have been in follow-up for years, but I see that things haven’t changed. On the contrary, it’s getting worse and worse.

parent 09

Parents expressed fear and anxiety related to exacerbations and, as identified in domain 1, showed different perceptions of the severity of their child’s asthma. These were identified as barriers to asthma self-management. Another barrier is poor adherence secondary to burden of medications. Discussions with the parents revealed that forgetfulness was often a specific cause of poor adherence, along with fear of side effects associated with medication intake.

Children and parents explored the role of SGs in the impact of asthma and barriers to asthma self-management, particularly the identification of real-life situations within the games. Specifically, children acquired *knowledge on self-management through games*. This theme is illustrated by the following quote from a child when talking about what they learned in the games:

Even if you are, for example, asthmatic, you can still do sports, but you have to take your pumps first before doing the sport.

child 05

Interestingly, children expressed being more aware of and able to focus on their breathing through the game, Bloïd, which uses a breath-actuated game controller. Similar themes were identified from the discussions with the parents, who identified acquired *knowledge on self-management through games* as a beneficial effect of the SGs. This included a better understanding of medication use and the potential of games to help in trigger recognition*.* This theme is exemplified in the following quote:

It [the SGs] was a practice even for me because I always mix up the blue and purple [pumps]. I ask myself ‘Which does what?’ And at a certain point [in the game], it was more visual for me and now the diagram is engraved in my brain.

parent 06

In addition, parents suggested that games may be particularly *helpful at the onset of asthma* and identified *games as a possible tool to evaluate asthma control* thereafter.

#### Domain 3: The Role of SGs in the Support System for Asthma Self-management

This domain includes different themes pertaining to the role of SGs in the child’s support system for asthma self-management, as perceived by children and their parents. Children expressed feeling alone with their asthma*,* whether it was among their peers or other family members. Children often refrained from discussing their condition with others. Parents expressed being supportive in their child’s asthma management, but this consisted primarily of the parents offering reminders to their children to take their medication. Often, parents were unaware of their child’s state of asthma knowledge and self-management.

Both parents and children identified the beneficial effects of SGs on their support systems for asthma self-management. Children highlighted how SGs could allow players to feel less alone with their asthma by increasing *asthma awareness through SGs.* In addition, the theme *openness to further discuss asthma with parents through games was* identified. This theme is illustrated by the following quote from a child when asked whether they would like to play the games with their parents:

Maybe they would learn more with us. Most of the time, they are the ones that give me advice because they listen to the doctors more. They know a little more, because even if they aren’t doctors, they know more about this subject. But we could interact at home and talk about what we thought [about the games].

child 09

A similar theme, *importance of parent and child playing together*, was also extracted from the discussions with the parents. This theme is illustrated by the following quote from a parent:

I don’t really play video-games, but I still learned things, not that I didn’t know, but that maybe I hadn’t absorbed. By playing, watching and participating with my daughter, I learned certain things.

parent 06

Parents appreciated the parent-child collaboration through gameplay, as it allowed them to be more aware of their child’s asthma self-management skills. Both children and their parents separately identified an *interest and utility of playing the games with peers*. Children showed a desire to interact with peers about their illness, whereas parents expressed the importance of sharing knowledge and playing with others as an incentive to learn and increase awareness among children without asthma.

## Discussion

### Principal Findings and Comparison With Previous Work

In this pilot study, we conducted qualitative individual interviews and group discussions with participants and their parents to evaluate the perceived role of SGs in asthma management and knowledge transfer achieved by these games. Our study identified the following domains, or components, that are key to asthma management: asthma self-management perception and knowledge, the impact of asthma and barriers to asthma self-management, and support system for asthma self-management. Within these domains, our team consensually identified various themes pertaining to the perceived role of SGs in the given components. In the following sections, we summarize our findings by identifying an overarching issue for each domain and explaining how SGs can be used to address this issue. The results are summarized in Figure 1.

In the domain of asthma self-management perception and knowledge, the heterogeneity of asthma knowledge, which is related to asthma perception, was identified as the overarching issue for both children and parents. The discussions with the children and parents revealed that some participants had adequate knowledge about asthma, including trigger recognition and different medications, whereas other participants had poor understanding of asthma and even expressed several false beliefs. Specifically, some children were unaware of resources other than the inhalers that were available to them, such as books or educational websites. Several parents not only expressed being in denial of the severity of their child’s condition but also displayed numerous misconceptions about asthma. The importance of health literacy, the capacity to understand and manage one’s own health, is a component emphasized in the eCCM and is essential for the proper management of chronic diseases and the adequate use of eHealth services [14]. The importance of efficient asthma education is equally emphasized in a systematic review of SGs, stating that education is an important factor in treatment because it promotes considerable ameliorations in asthma control and reduces emergency room visits and hospital admissions [15]. To address this heterogeneity in knowledge, the children and parents expressed that SGs could improve knowledge transfer through games*.* Indeed, as highlighted by a comparative study between asthma educational videotapes and asthma self-efficacy digital games, interactive media with active participation are more effective in improving self-management than traditional teaching methods such as educational videos [32]. Moreover, our participants appreciated the real-life scenarios presented in the games and expressed that such animations would provide them the knowledge to react accordingly in different situations. Similarly, parents considered the games to be a valuable source of information to build a strong base for a child with limited knowledge about asthma. These findings corroborate with those of other studies that have demonstrated how traditional educational methods have little appeal to children and the importance of using narratives as a novel educational tool [16,33]. Narratives have been shown to be a useful resource in promoting a great understanding of the impacts an illness can have and providing an emotional insight into the repercussions a disease can cause [33].

Within the domain pertaining to the impact of asthma and barriers to asthma self-management, the consequences and limitations of asthma surfaced as the overarching issue for both children and their parents. Despite having access to regular medical care, children and parents expressed being subject to the consequences of the child’s asthma, specifically regarding activity limitation and fear and anxiety related to asthma exacerbations. According to the eCCM, having a patient who is empowered and in control of their illness is crucial to improve health outcomes [14]. The eCCM also emphasizes that a patient must be *activated*, a term that equates to an individual’s level of skills, knowledge, and confidence in managing their disease [14]. As highlighted in our study, the model of a patient who is activated and empowered should also be extended to a child’s caregiver in pediatric care, given that caregivers play an essential role in their child’s treatment [15]. Although barriers to asthma self-management have been extensively discussed previously [12], we wanted to focus on how SGs could facilitate asthma self-management. Children and their parents expressed that SGs could provide an opportunity for the identification and management of real-life situations through games. SGs can aid in promoting self-management and patient empowerment through the social learning theory, which suggests that through self-modeling (ie, the players observe a character in the game who is a representation of themselves and who they control), the player is able to learn through his or her character and apply what they have learned to real-life situations [17]. In addition, a study concerning the application of health games to manage chronic pediatric diseases showed that, often, children with chronic health conditions experience low self-esteem and stigmatization from peers because of daily self-care and monitoring [32]. However, by presenting characters who have the chronic disease and who represent positive role models who achieve their missions in the SGs while simultaneously battling and managing their illness, children learn that their chronic disease can be overcome and that self-management is an achievable goal [32].

Finally, in the domain related to support system for asthma self-management, we identified an insufficient support system as the overarching issue among children and parents. In the case of children, family implication in the child’s condition is essential, an idea that was equally mentioned by the children in our study, who viewed their parents as a source of support. Notably, effective parent-child collaboration in managing a child’s asthma is a key element in improving medication adherence and positive health outcomes [7]. Children and parents expressed that SGs could provide an opportunity to play with others for support and shared knowledge. Indeed, both parents and children expressed an interest in playing the SGs presented in this study with each other, stating that this would also allow for more parent-child discussions about the condition. A study concerning the management of chronic pediatric diseases with health games found that 1 month after playing asthma self-management games, the children experienced more self-efficacy in talking with their friends about asthma and showed increased communication with their parents about their condition, illustrating how SGs can help strengthen a child’s support system [32]. Similarly, an inpatient study evaluating the impact of asthma self-management SGs revealed that, often, during gaming sessions, one child would explain asthma management strategies to another child, allowing both players to win the game together [32]. Therefore, SGs offer a unique, interactive, and enjoyable opportunity for children with asthma to learn and interact with their peers and parents.

### Strengths and Limitations

Our study has numerous noteworthy strengths and limitations. First, compared with previous studies in the field, the originality of our study stems from the inclusion of both children and their parents in the gaming sessions and discussion groups, allowing for the comparison and contrast of thoughts and perceptions. This is particularly important given the unique parent-child interactions in pediatric chronic disease management. Second, we based the development of our games on the theory of co-design, adapting our games based on the comments and input from users throughout their development. Thus, the versions presented to participants in this study have already taken the input of patients with asthma into account, making them even more pertinent to the study population. Third, the analysis of our qualitative data was achieved through consensual qualitative research, a research method that incorporates various validation methods to ensure the validity of the results (ie, member checking and discussing until agreement was reached).

However, despite these validation methods, a possibility of subjectivity in our results remains, emphasizing the need for more extensive member checking. The small sample size and inclusion of patients followed at a pediatric hospital may reflect potential selection bias and limit the generalizability of our results. Specifically, the feedback gathered from the limited sample of children may not reflect the perspectives of other children with asthma. In addition, owing to the limited number of participants, we were unable to conclude any statistically significant changes in terms of pregaming and postgaming knowledge from the participant questionnaires. Thus, a large study is needed to better evaluate the impact of these SGs on asthma knowledge and to collect additional feedback from players. Given the design of the study, the time allotted to play was limited to a total of approximately 60 minutes, which may have affected the depth of the evaluation of the game by participants. Although we were able to gather valuable feedback, further studies are needed to evaluate the acceptability of the games in different settings (eg, at home or during free play).

### Continued Development of the SGs

Following this study, feedback from children and parents was integrated into the games, Asthma Heroes and Asthmonautes. Both games were translated and are now accessible in French, English, Spanish, Chinese, Russian, Arabic, Portuguese, Japanese, Vietnamese, Korean, Farsi, Turkish, German, and Italian. In addition, our team conducted a second study, where 158 children tested a game played using a breath-activated controller, which enables the assessment of one’s breathing capacity [34].

### Conclusions

In conclusion, through discussions with children with asthma and their parents and consensus within our research team, we identified various themes pertaining to how SGs can address some of the perceived barriers related to asthma self-management. Although our pilot study was based on a limited number of participants and further studies are required to confirm our results, our findings support the acceptability of SGs by both children and their parents and their potential role in asthma education and self-management. The numerous potential benefits of SGs in various aspects of asthma management highlight the necessity for future developments in this field.

## Abbreviations

CCM: Chronic Care Model
eCCM: eHealth Enhanced Chronic Care Model
SG: serious game
